# Fluidically Switchable Metasurface for Wide Spectrum Absorption

**DOI:** 10.1038/s41598-018-28574-9

**Published:** 2018-07-05

**Authors:** Saptarshi Ghosh, Sungjoon Lim

**Affiliations:** School of Electrical and Electronics Engineering, Chung-Ang University, Heukseok-Dong Dongjak-Gu, 156-756 Republic of Korea

## Abstract

Metasurfaces, owing to their attractive features, provide a wide range of potential applications. Electromagnetic absorbers based on metasurfaces have significantly improved responses compared to the earlier absorbers made from composite materials. Active metasurfaces, in contrast to the passive designs, can exhibit multifunctional characteristics without repeated fabrication. This paper presents a fluidically-reconfigurable active metasurface that provides switchable wide spectrum absorption. The proposed design is comprised of liquid-metal-encased dielectric substrates, sandwiched between the top resistive pattern and bottom ground plane. With precise control of the liquid metal flow, the structure can exhibit wide absorption bandwidth switching between two frequency regimes. Further, the proposed metasurface has a significant advantage of displaying polarization-insensitive behaviour, unlike the previous fluidically-reconfigured structures. The design has been investigated by illustrating surface current distributions and several parametric variations. Finally, the proposed structure was fabricated using laser etching, and experimentally validated. This work has paved the way towards the realization of reconfigurable metasurfaces with multifunctional characteristics, thus showing great potential in microfluidic technology for diverse applications.

## Introduction

Electromagnetic (EM) absorption is a widely nurtured phenomenon across research fields, owing to its manifold applications, such as radar cross section reduction, EM compatibility, EM interference, stealth technology, imaging devices, and wireless communication^1–4^. Conventional absorbers, mostly made of composite materials, suffer from bulk thickness and fragile behaviour, at the expense of large weight and high cost^5–8^. Two-dimensional (2D) metamaterials, also considered as metasurfaces, have gained significant research interest since their conceptual realization and experimental demonstration^9,10^. Owing to their attractive features, metasurfaces have been exploited in absorber technology^11,12^ and were found to have improved responses in terms of ultra-thin thickness, small weight, and near-unit absorptivity compared to the existing absorber structures. With the advent of techniques, narrow-band^13–15^ as well as broadband^16–19^ metasurface-based absorbers have been designed, possessing various characteristics.

However, most of the earlier metasurfaces are built on passive geometries, which perform single functionality without repeated fabrication. With the growing demand in the EM spectrum, active metasurfaces capable of exhibiting dynamic behaviours have been extensively pursued^20–22^. These adaptive metasurfaces, through controlling the external stimuli, offer multifunctional characteristics to mitigate the issues above^23–25^. Tunable geometries regulate the resonances in a particular state^26–28^, whereas switchable structures vary the responses between different states (absorption, reflection, transmission) at the frequency of interest^29^. A switchable metasurface has been presented where the response gets shifted between perfect reflection and narrow-band absorption by the use of a PIN diode^30^. Switchable absorbers, based on their requirements, exhibit absorption performances that can be shifted discretely between two particular frequencies (narrow-band)^31,32^ or two wide spectrum (broadband)^33^. The absorption spectrum gets switched between different frequency ranges, owing to the switching activity of the active components^34^. Nevertheless, modulation of the broadband absorption with reconfigurable feature is indeed difficult, and often requires in-depth studies.

Since the past decade, several techniques have been attempted to realize adaptive metasurfaces. Electrically actuated materials, such as ferrite substrates^35^, and composite polymers^36^ have been investigated; however, they suffer from large array fabrication difficulty and weak modulation characteristics. Graphene can be used to realize adaptive metasurfaces through controlling the charge density, but the techniques are still challenging for realistic device configurations^37,38^. Active circuit elements (diodes, transistors) integrated with passive metallic structures are also implemented; however, the external biasing circuitries further complicate the designs^39–41^. Recently, microfluidic technology has been exploited to develop active metasurfaces that do not rely on the electronic devices or complicated fabricated procedures^42–46^. In this technique, microfluidic channels are engraved in an elastomeric substrate through laser etching. Using the required pressure, liquid metal (or metal alloy) slugs can flow through the channels, thus regulating the frequency response. Liquid metals, owing to their self-healing property, can adopt any arbitrary shape based on the microfluidic channel geometry^47,48^. Additionally, they can exhibit switchable conductivity via external control, thus offering a good alternative in adaptive metasurfaces in the microwave regime. Recently, a microfluidically-reconfigured switchable broadband absorber has been demonstrated based on the coupling between the top metallic geometry and bottom liquid-metal-filled channels^43^. However, the proposed structure suffers from polarization-sensitive behaviour, and exhibits small absorption bandwidth.

This article presents a switchable metasurface for wide spectrum absorption by leveraging the properties of a fluidically reconfigurable technique. The proposed geometry has the novelty of exhibiting broadband absorption (over 90%) that switches from C to X band (in the microwave range), with the injection of liquid metal in the microchannels. The design has another notable characteristic of having four-fold symmetry (thus showing polarization-insensitive behaviour) over the earlier reported switchable structures. Angular stability analysis as well as the study on several parametric variations have been pursued to explore the salient features of the geometry. Experimental verification has further confirmed the numerical simulation of the proposed structure. Broadband switchable absorption, mechanical flexibility, along with polarization-insensitivity have rendered the proposed design a unique configuration for the realization of adaptive microwave metasurfaces.

## Results

### Numerical Simulation

**Figure 1:**
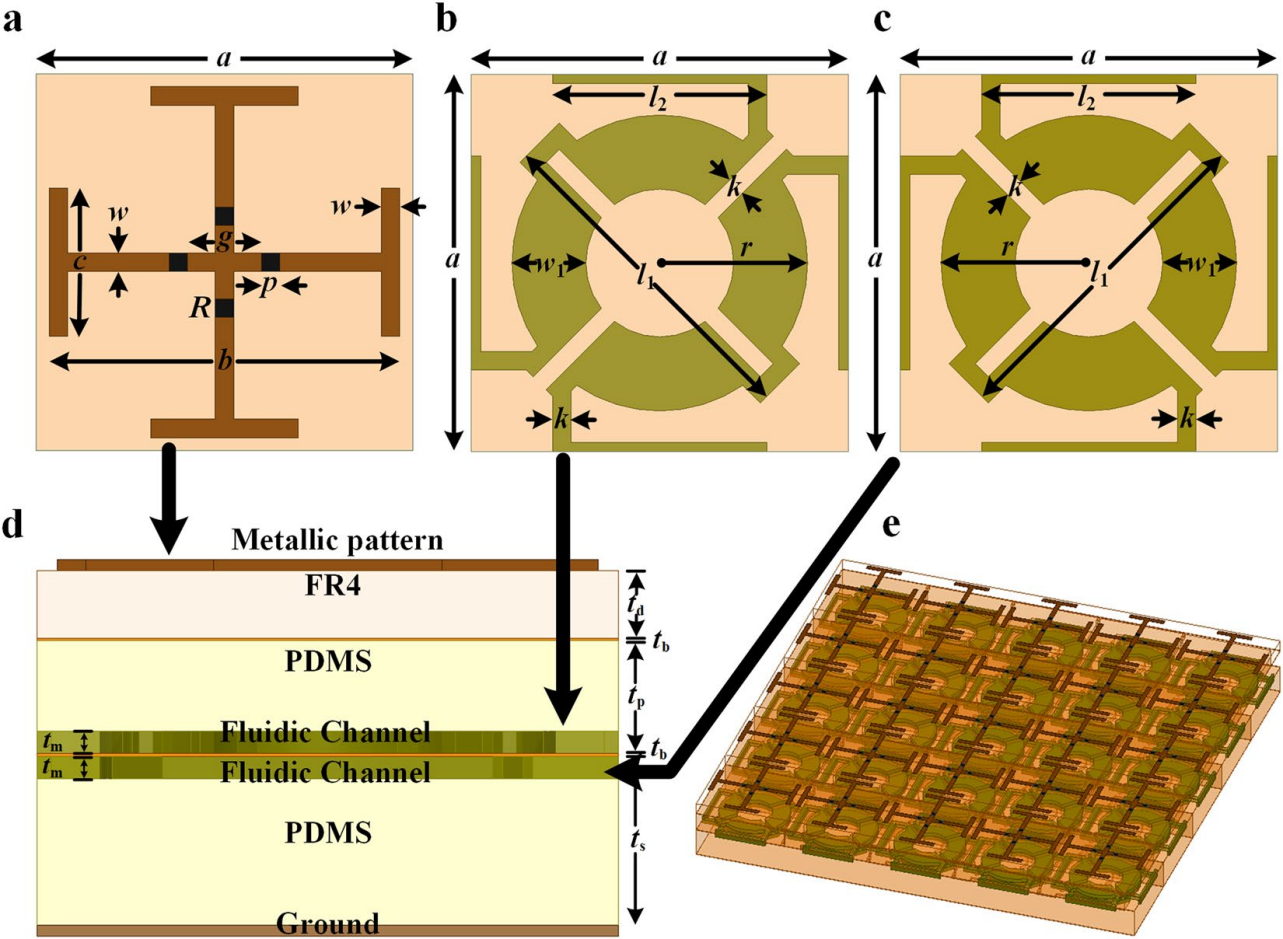
Proposed Switchable metasurface for wideband absorption. (**a**) Top view of top metal-imprinted-FR4 dielectric layer. The dimensions of the unit cell geometry are: *a* = 10.4 mm, *b* = 9.4 mm, *c* = 4 mm, *g* = 2 mm, *p* = 0.8 mm, *w* = 0.5 mm, and *R* = 100 Ω. (**b**) Top view of middle layer comprising microchannel-inscribed-PDMS layer, where *l*_1_ = 9.2 mm, *l*_2_ = 5.8 mm, *w*_1_ = 2 mm, *r* = 4 mm, and *k* = 0.5 mm. (**c**) Top view of bottom layer consisting of microchannel-inscribed-PDMS layer, with identical parameters. (**d**) Side view of the overall metasurface, resulting from stacking multiple layers (*t*_d_ = 1.2 mm, *t*_p_ = 3 mm, and *t*_s_ = 2 mm). Individual layers are joined together using thin bonding film (*t*_b_ = 50 μm), whereas the microfluidic channels are engraved inside the PDMS layers with precise thickness (*t*_m_ = 0.4 mm). (**e**) Perspective view of the periodic array structure.

Figure 1 shows the schematic of the proposed switchable metasurface that comprises three vertically stacked metal-dielectric layers. The top layer, as depicted in Fig. 1a, is a metal-imprinted-dielectric substrate, where lumped resistors are mounted symmetrically across the gaps in the metallic pattern. Copper (*σ* = 5.8 × 10^7^ S/m) is used as the metal, and FR4 is chosen as the dielectric substrate (*ε*_*r*_ = 4.4, and *tan δ* = 0.02) for the top layer. The middle and bottom layers, as shown in Fig. 1b,c, respectively, are made of flexible polydimethylsiloxane (PDMS) substrates, where liquid metal is encapsulated inside the microchannels. One set of fluidic channels is engraved at the bottom side of the middle PDMS layer, whereas the other set of microchannels are inscribed on the top of the bottom PDMS layer, as indicated in Fig. 1d. A thin bonding layer is introduced between the consecutive layers, primarily for two reasons: first, to join the dielectric substrates without any intermediate spacing, and second, to enclose the liquid metal slug inside the microchannels. Eutectic gallium indium (EGaIn), a commercially available liquid metal alloy, has been used in the design. The alloy has a composition of 75% Ga and 25% In by weight, which offers low viscosity along with minimal toxicity. The microchannels are diagonally connected across the unit cells such that EGaIn can flow continuously. The periodic array of the proposed design is illustrated in Fig. 1e.

Metasurfaces, on the incidence of the plane EM wave, exhibit reflection, transmission, and absorption, while satisfying the equation^11^: *A* = 1 − |*S*_11_|^2^ − |*S*_21_|^2^. Since the structure is laminated by the metal ground, transmission does not take place; hence, absorptivity can be resulted by the impedance matching (|*S*_11_| = 0) of the design. The proposed metasurface, comprising a resistor-embedded metallic pattern in the top layer, absorbs the incident energy over a wide spectrum^49,50^. This broadband absorption also depends on the overall substrate thickness of the structure. The larger the thickness, the more redshift is the absorption bandwidth. This concept has been exploited in our proposed design to achieve the switching phenomenon in the absorption spectrum.

**Figure 2:**
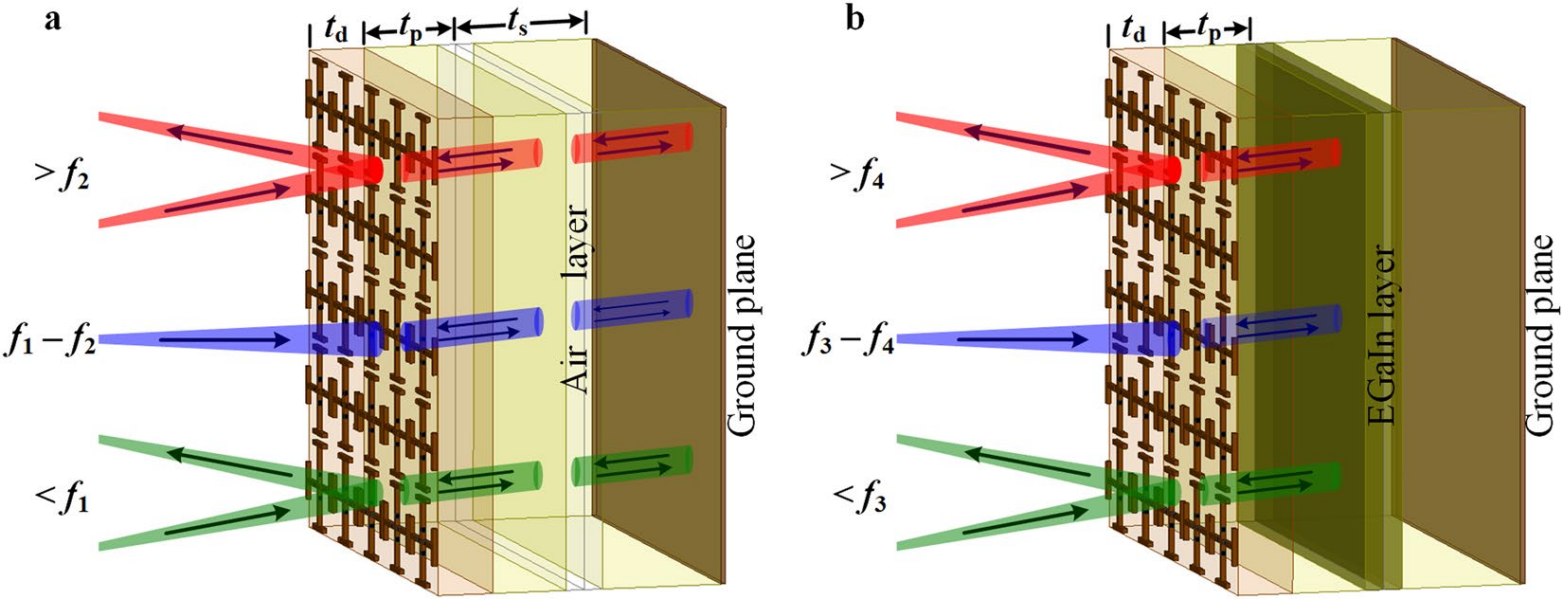
Three-dimensional illustration of the proposed switchable metasurface arranged in periodic array. (**a**) When there is no liquid metal inside the microchannels, incident EM wave travels through the entire thickness and broadband absorption occurs over the frequency range of (*f*_1_ – *f*_2_). (**b**) With the injection of EGaIn, the microchannels behave as an adaptive ground, and restricts EM wave for further penetration, which causes the shift in the absorption spectrum to (*f*_3_ – *f*_4_). Outside the absorption window, the incident wave is reflected back to the first medium, as indicated by the red and green arrows. Absorption of EM wave is designated by the blue arrows.

For broadband absorption to be operated in a low frequency range, the microchannels are made completely empty, i.e., filled with air. Subsequently, the structure has an effective height of *t*_d_ + *t*_p_ + *t*_s_ (ignoring the bonding layer thickness). This results in a broadband absorption over the frequency range of *f*_1_ – *f*_2_, as illustrated in Fig. 2a. On the contrary, the effective height reduces to *t*_d_ + *t*_p_, with the injection of liquid metal inside the microchannels. When EGaIn is inserted inside the channels, the incident EM wave is reflected from this layer only, without further penetrating through the bottom PDMS layer (of thickness *t*_s_). Thus, the effective height becomes reduced; consequently, the absorption spectrum shifts to the higher frequency regime of *f*_3_ – *f*_4_, as shown in Fig. 2b. With the removal of EGaIn, the substrate thickness returns to its earlier condition, and the broadband absorption is reinstated in the lower spectrum.

**Figure 3:**
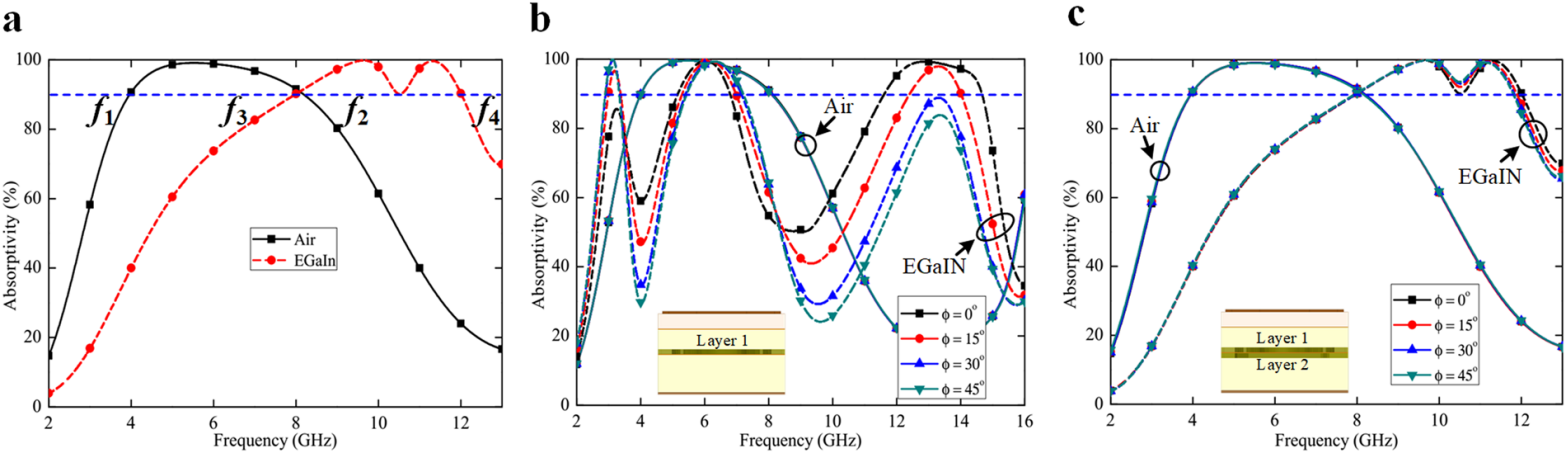
Simulated response of the proposed metasurface. (**a**) Simulated absorptivity of the structure under normal incidence. (**b**) Simulated absorptivity of the design for different polarization angles, with single microfluidic channel in the PDMS layer. (**c**) Simulated absorptivity of the proposed structure for different polarization angles, with two microchannels positioned in close proximity in the PDMS layers. The dashed and solid lines represent the responses with and without EGaIn injection, respectively.

Figure 3a shows the simulated absorption response of the proposed structure for two different switching states, with and without the injection of EGaIn. Without the metal alloy, the design exhibits broadband absorption above 90% over the range of 4.02–8.20 GHz, whereas the bandwidth shifts to a higher side (8.04–12.04 GHz) with the inclusion of EGaIn. The design has been optimized to cover the individual microwave spectrum in two different states, i.e., C-band during air-filled channels, and X-band under EGaIn-filled channels. The absorption bands during EGaIn-filled-channels are slightly smaller (in terms of fractional bandwidth) compared to its counterpart, since the design has been optimized to cover two separate microwave bands during two different switching states.

The proposed structure has the unique topology that utilizes two EGaIn-filled microchannels, positioned in close proximity with orthogonal orientation. This particular alignment provides two significant advantages to the design as compared to our previous study^43^: first, the absorption spectrum becomes widened, and second, the design becomes polarization-insensitive. With single metal-filled microchannels (as illustrated in Fig. 3b), the absorption bandwidth is narrower and the switching performance is unsatisfactory. Further, the metasurface exhibits polarization-sensitive behaviour, due to the asymmetry in the microchannel geometry. In contrast, the proposed design (with the inclusion of two orthogonally oriented microchannels) presents a wider absorption spectrum, discrete switching response, as well as polarization-insensitive characteristics, as observed in Fig. 3c. Although a single-layer microfluidic channel with four-fold symmetry might exhibit polarization-independent properties, this type of channel geometry cannot be realized to inject and/or extract the liquid metal monotonically. Thus, the fluidic channel has been divided between two layers, with orthogonal arrangement. Besides, the patterns should be continuous across the unit cells, such that liquid metal alloy can be monotonically injected or extracted through peripheral control. The circular loops have also been considered to avoid any sharp bending in the metallic pattern, such that there will be no liquid metal leakage from the channels. The interconnecting lengths between the microchannels across the neighboring unit cells might be further contracted, but that would break the four-fold symmetry. Then, the design would not behave as polarization-sensitive, unlike our previous switchable absorber^43^.

**Figure 4:**
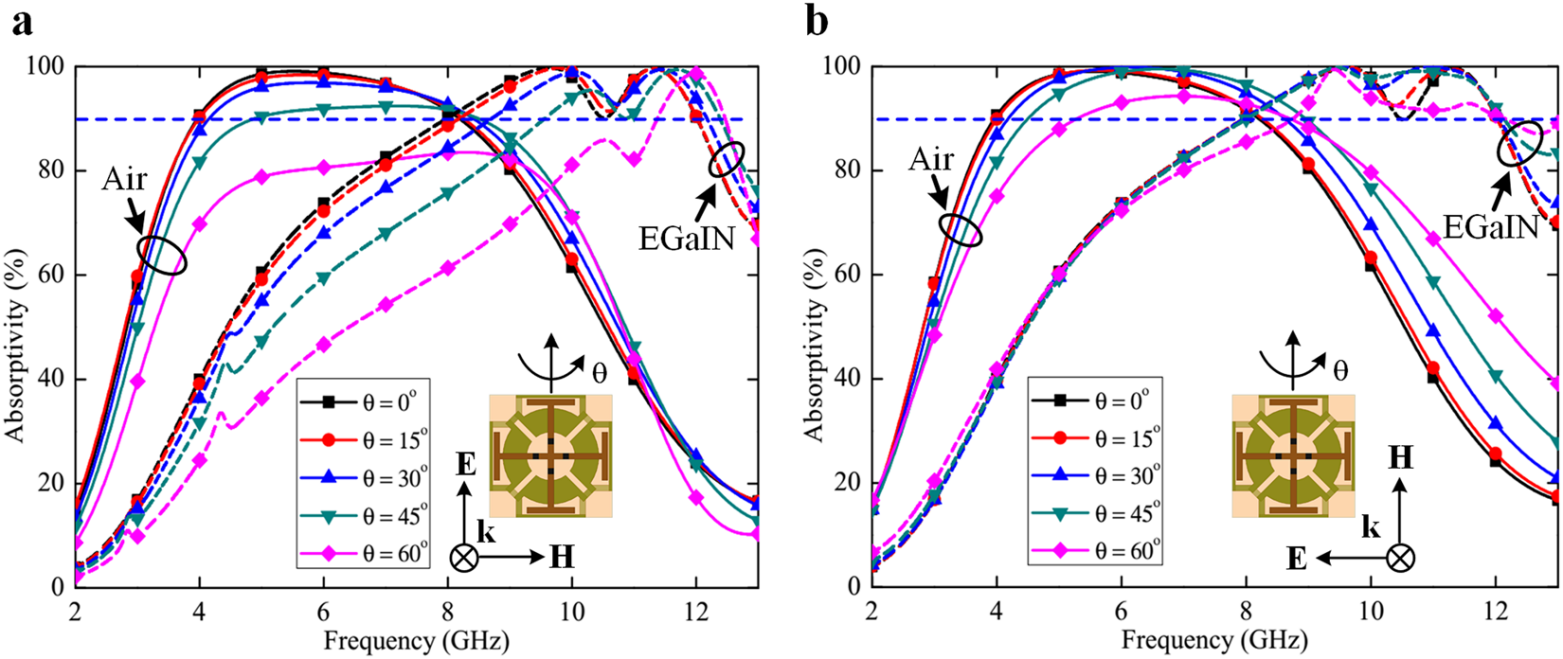
Simulated absorptivity of the proposed metasurface under oblique incidence. (**a**) Simulated absorptivity under different incident angles for TE polarization. (**b**) Simulated absorptivity under different incident angles for TM polarization. The dashed and solid lines represent the responses with and without EGaIn injection, respectively.

The proposed structure has been examined under oblique incidence and found to be angularly stable, under both transverse electric (TE) and transverse magnetic (TM) polarizations. In TE polarization, the incident electric field remains in the transverse direction with the structure interface, while the incident magnetic field and the wave propagation vector directions are rotated through an angle of theta (*θ*). On the contrary, the incident magnetic field remains constant during TM polarization, whereas the incident electric field and the propagation vector directions are varied by the angle. Figure 4a shows the absorption response of the proposed design for TE polarization, where the structure exhibits broadband absorption (above 90%) until 45° incident angle. Under TM polarization, the absorptivity still exists at 60° angle of incidence for both switching states, as depicted in Fig. 4b. Broadband absorbers with angular stability is much required in practical applications, and the proposed metasurface is a high-potential candidate owing to its commendable performance.

**Figure 5:**
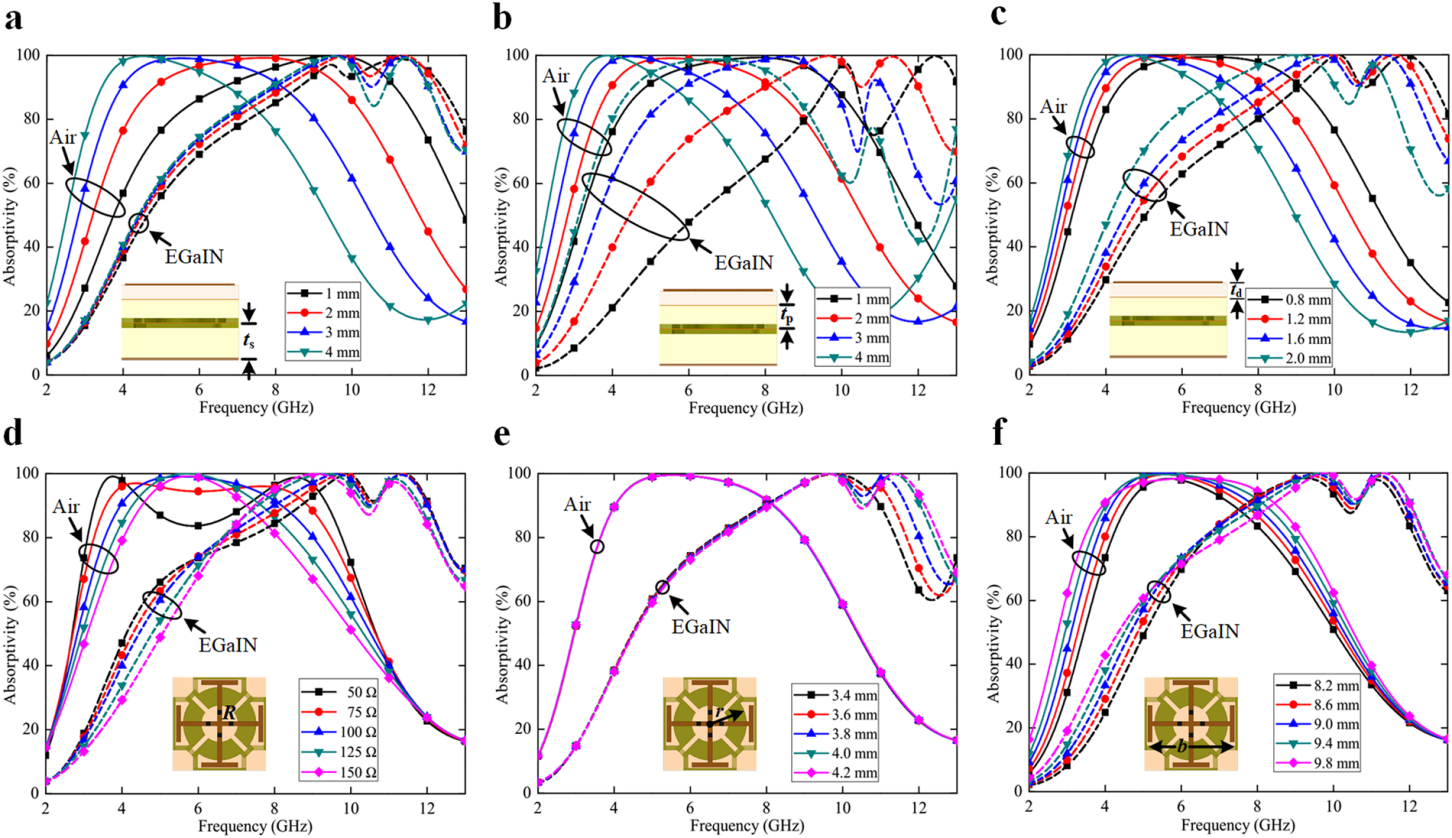
Simulated absorptivity of the proposed metasurface for various parametric variations, under different switching states. (**a**) Variation in the bottom PDMS layer thickness (*t*_s_). (**b**) Variation in the middle PDMS layer height (*t*_p_). (**c**) Variation in the top FR4 dielectric substrate thickness (*t*_d_). (**d**) Variation in the lumped resistor value (*R*) mounted across the gaps in the top metallic pattern. (**e**) Variation in the radius of the microchannels (*r*) engraved inside the PDMS layers. (**f**) Variation in the length of the metallic pattern (*b*) imprinted on FR4 substrate. The dashed and solid lines represent the responses with and without EGaIn injection, respectively.

To further elucidate the broadband absorber, several parametric variations have been investigated to explain the switching characteristic of the design. When the height of the bottom PDMS layer (*t*_s_) is gradually raised, the wide spectrum absorption shifts to a lower frequency range for a particular switching state (when channels are air-filled), as illustrated in Fig. 5a. However, no significant deviation is observed corresponding to the other state, while EGaIn is encapsulated inside the microchannels. The EGaIn-filled channels behave as a switchable ground plane, and thus the PDMS layer present beneath the fluidic channels contribute little effect to the absorption performance.

However, the effective dielectric thickness gets modulated for both switching states, with the variation in the top PDMS layer thickness (*t*_p_). While increasing the height, the absorption bandwidth progressively shifts to a lower frequency regime, as shown in Fig. 5b. Similarly, alteration in the top FR4 substrate thickness (*t*_d_) results in the analogous response, as observed from Fig. 5c. These parameters can be precisely regulated to control the extent of switching between the absorption bandwidths.

With the increase in the lumped resistor value (*R*), the absorption peaks gradually move towards each other, thereby resulting in higher absorptivity, but at the expense of smaller bandwidth. Figure 5d exhibits the variation in the absorption spectra of the proposed metasurface while the value of the lumped resistors is changed. The figure also confirms that the broadband absorption is mostly caused by the ohmic loss resulting from the lumped components, rather than the dielectric loss of the substrates.

Figure 5e depicts the effect in the absorptivities corresponding to the variation in the radius of the microchannels (*r*) of the proposed design. When the fluidic channels are air filled, no significant modulation in the absorption response is observed. However, with the inclusion of EGaIn in the microchannels, the absorptivity gets improved with the larger radius. Finally, when the length of the metallic pattern (*b*) is enhanced, the absorption band shifts to the lower frequency range, for both switching states, as observed in Fig. 5f. The larger the length of the geometry, the wider is the shift in the absorption spectrum.

**Figure 6:**
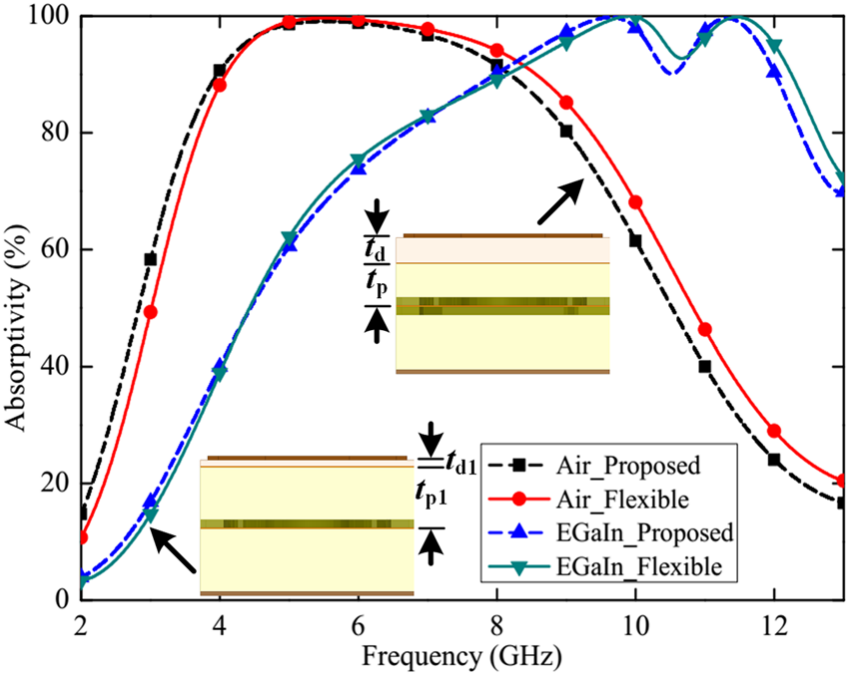
Comparison of simulated absorptivities between the proposed metasurface and the flexible one. The geometric dimensions of the proposed design and the flexible structure are almost identical, except the two parameters: *t*_d_ = 1.2 mm, *t*_p_ = 2 mm (in the proposed one), and *t*_d1_ = 0.2 mm, *t*_p1_ = 3.2 mm (in the flexible one).

The top FR4 dielectric has primarily been selected due to its low cost and commercial availability. A soft dielectric material can replace the rigid FR4 substrate, thus offering an extra degree of control over the absorption bands by bending/stretching the metasurface. Even, FR4 substrate with thin profile can also serve the purpose of displaying flexible characteristic. The proposed design has been modified by replacing the rigid FR4 substrate (*t*_d_ = 1.2 mm) with an ultrathin flexible FR4 sheet (*t*_d1_ = 0.2 mm). At the same time, the middle PDMS layer thickness has been increased from 2 mm (*t*_p_) to 3.2 mm (*t*_p1_), to maintain the same absorption profile. Figure 6 shows the comparison of the simulated absorptivities between the proposed structure and the flexible one. It is clearly observed that with the use of a flexible dielectric, the design is still able to provide similar switchable absorption characteristic.

### Experimental Verification

**Figure 7:**
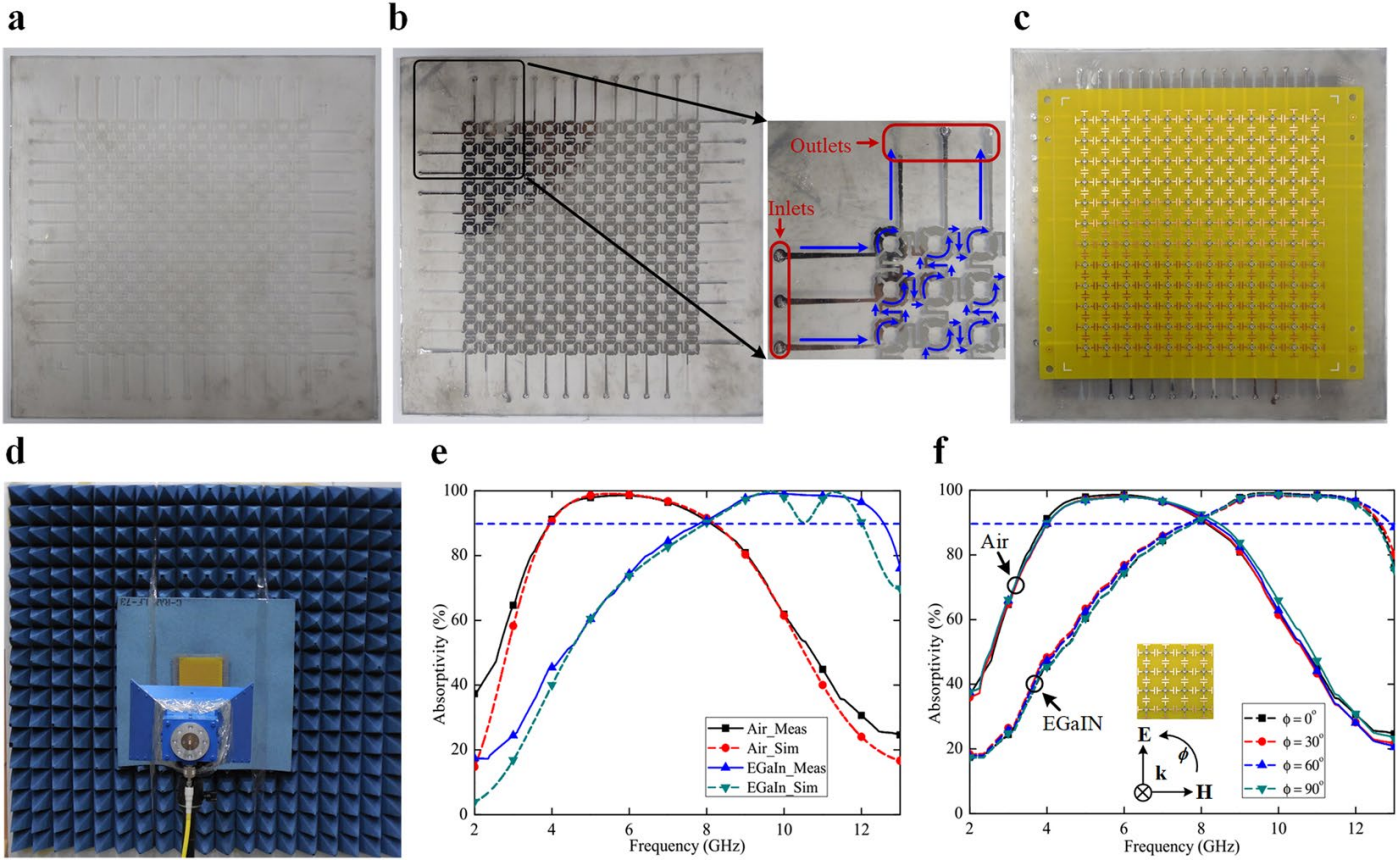
Experimental verification of the proposed metasurface. (**a**) Photograph of the PDMS layer without EGaIn injection. (**b**) Photograph of the PDMS layer with EGaIn injection. Insets show the zoomed views of the sample with of the flow of EGaIn. Liquid flow (shown in blue line) is bidirectional; only one way is shown for clarity. (**c**) Photograph of the complete fabricated prototype. (**d**) Measurement set-up. (**e**) Comparison of the measured and simulated absorptivities of the proposed metasurface under normal incidence. (**f**) Measured absorption responses of the fabricated sample for different polarization angles, under different switching conditions.

To experimentally demonstrate the switchable metasurface, a prototype was fabricated using hybrid techniques (see Methods for the detailed fabrication steps). The sample comprises 12 × 12 unit cells, thereby having a total dimension of 124.8 mm × 124.8 mm. The PDMS layers, without and with the inclusion of liquid metal EGaIn, are depicted in Fig. 7a,b, respectively, along with their zoomed views. The complete fabricated prototype, realized through stacking the PDMS layers and the resistor-loaded substrate, is illustrated in Fig. 7c.

The microchannels are designed in such a way that the neighboring unit cell patterns are connected with one another diagonally. Each diagonal is equipped with one set of inlet and outlet, as illustrated in the inset of Fig. 7b. Inlets are used for EGaIn injection, whereas extraction of the liquid metal can be made through the outlets.

The dynamic control of liquid metal (alloy) in the microchannels can be modulated through various methods. In electrocapillarity technique, a low voltage is applied between the inlet and outlet of the microchannels, and the liquid streams efficiently without any mechanical stimuli^51^. However, the metal has a tendency to break up into droplets through interfacial instabilities and therefore is not preferred in our configuration. Electrowetting technique is also used in certain devices (such as EM polarizer), but the actuation is mostly limited to the change in the contact angle of the conductive fluid, rather than controlling the flow^52^. Therefore, the liquid metal alloy was inserted into the microfluidic channels by the use of a syringe. It took approximately 2–3 minutes to fill all the microchannels; however, the injection time can be significantly reduced by the use of a piezo-actuator-based micropump. Outlets were used to extract the liquid from the prototype. Because of single-inlet-single-outlet concept, there was no difficulty in reconfiguring the sample, irrespective of the number of repetitions.

The sample was measured using the free space technique (see Methods for the measurement procedure)^53,54^, facilitated by a broadband antenna and a network analyzer. When no EGaIn was injected inside the microchannels, the prototype exhibited broadband absorption (above 90%) over the range of 2.48–8.10 GHz. However, the absorption spectrum shifted to 7.98–12.24 GHz after the insertion of EGaIn inside the microchannels. When the liquid was further extracted through the outlets, the metasurface reinstated into its previous state, and the absorption band returned to its previous position. The measured response was also compared with the simulated results (see Fig. 7e), and was found to be in good agreement for both switching states. Although some minor deviations were observed, in particular away from the absorption peaks, they might be attributed to the fabrication tolerance and parasitic effects of the lumped components.

Since the microfluidic technique is a delicate one, several precautions need to be considered during measuring the prototype. EGaIn gets easily oxidized when comes in contact with oxygen (air) and tends to leave a residue on the substrate^55^. This may prevent the metasurface from repetitive use. Therefore, in the sample, the microchannels were covered with bonding films (ARcare®92561) and air was removed from the channels with utmost care. Several other solutions, such as – use of Teflon solution^56^, coating with Hydrocal liquid^57^ can also be used to avoid any direct contact between EGaIn and air. Liquid metal spray printing^58^, although fast and convenient, cannot be used in such microfluidically-reconfigurable metasurfaces owing to its possible contamination with air during spraying.

To validate the polarization-insensitive characteristic of the adaptive metasurface, the sample was also measured at different polarization angles under normal incidence. Figure 7f indicates the absorption responses of the structure and shows similar spectra for all the polarization angles, under different switching conditions. Thus, the polarization independence of the switchable metasurface has also been experimentally demonstrated.

## Discussion

In summary, we demonstrated a novel class of switchable metasurface, designed for wide spectrum absorption. The proposed structure exploits the property of encapsulating liquid metal in the microchannels, which can be precisely actuated to regulate the absorption bandwidth between potential microwave bands. With the injection of EGaIn slug inside the channels, an adaptive ground plane has been realized, thereby reducing the effective substrate thickness of the geometry. Since the liquid metal alloy needs to be flown uninterruptedly across the unit cells, the microchannels have the restriction of having to design asymmetrical geometries. This limitation has been resolved in the structure through constructing two orthogonally oriented fluidic channels connected in close proximity, which results in the polarization-insensitive characteristic. This particular topology has not been studied previously, to the best of our knowledge, and might stimulate the microfluidic technology for further use in practical applications. The absorption mechanism of the proposed metasurface has also been in-depth analyzed in this article through deriving several parametric variations. Switchable absorption bandwidth, coverage of potentially active microwave frequency bands, polarization-insensitivity, and the utilization of liquid metal alloy as switchable ground plane might pave the way for adaptive metasurfaces in the near future. Further investigations may be conducted to explore various kinds of modulating devices along with multifunctional characteristics that could find immense applications in radar technologies.

## Methods

### Full-wave simulation

A full-wave electromagnetic simulation of the proposed metasurface was performed in a finite element method (FEM)-based commercial software ANSYS High Frequency Structure Simulator (HFSS). A single unit cell was numerically simulated using periodic boundary conditions. The floquet port was used for the excitation of the geometry, whereas master-slave boundary conditions replicated the unit cell into a periodic array structure. The electrical characteristics of the substrates and the metals were obtained either from the simulation library or the available datasheets.

### Metasurface fabrication

The proposed switchable metasurface was fabricated using hybrid techniques. The top metallic geometry was patterned on an FR4 substrate using printed circuit board etching, on which lumped resistors were soldered using surface-mount technology. Laser etching was utilized to engrave the microfluidic channels inside the PDMS substrates. The dielectric substrates were joined with one another using thin bonding films, which were also utilized to encapsulate the liquid metal inside the microchannels. After fabricating the prototype, EGaIn was injected inside the channels to obtain the switching characteristic. Because of the unique channel configuration, the liquid can seamlessly flow across the unit cells, and the sample can be reconfigured without difficulty, irrespective of the number of repetitions.

### Measurement

The fabricated sample was measured in an anechoic chamber using the free space technique. A broadband horn antenna (1–18 GHz) was connected to the network analyzer (Anritsu MS2038C) to measure the reflection coefficient of the prototype. The measurement set-up is illustrated in Fig. 7d. Before conducting the measurement, the set-up was normalized by measuring the reflection coefficient from a copper plate of identical dimensions. Time gating was used to enhance the measurement accuracy as well as to reduce the background noise caused by multipath propagation.

### Data availability

All relevant data of this study are available within the article.
